# The effect of microvascular obstruction and intramyocardial hemorrhage on contractile recovery in reperfused myocardial infarction: insights from cardiovascular magnetic resonance

**DOI:** 10.1186/1532-429X-15-58

**Published:** 2013-06-27

**Authors:** Ananth Kidambi, Adam N Mather, Manish Motwani, Peter Swoboda, Akhlaque Uddin, John P Greenwood, Sven Plein

**Affiliations:** 1 Multidisciplinary Cardiovascular Research Centre & The Division of Cardiovascular and Diabetes Research, Leeds Institute of Genetics, Health & Therapeutics, University of Leeds, Leeds LS2 9JT, UK

**Keywords:** Hemorrhage, Cardiovascular magnetic resonance, Myocardial infarction, Strain

## Abstract

**Background:**

Following acute myocardial infarction (AMI), microvascular obstruction (MO) and intramyocardial hemorrhage (IMH) adversely affect left ventricular remodeling and prognosis independently of infarct size. Whether this is due to infarct zone remodeling, changes in remote myocardium or other factors is unknown. We investigated the role of MO and IMH in recovery of contractility in infarct and remote myocardium.

**Methods:**

Thirty-nine patients underwent cardiovascular magnetic resonance (CMR) with T2-weighted and T2* imaging, late gadolinium enhancement (LGE) and myocardial tagging at 2, 7, 30 and 90 days following primary percutaneous coronary intervention for AMI. Circumferential strain in infarct and remote zones was stratified by presence of MO and IMH.

**Results:**

Overall, infarct zone strain recovered with time (p < 0.001). In the presence of MO with IMH and without IMH, epicardial strain recovered (p = 0.03, p < 0.01 respectively), but mid-myocardial or endocardial strain did not (mid-myocardium: p = 0.05, p = 0.12; endocardium: p = 0.27, p = 0.05, respectively). By day 90, infarcts with MO had more attenuated strain in all myocardial layers compared to infarcts without MO (p < 0.01); those with IMH were attenuated further (p < 0.01). Remote myocardial strain was similar across groups at all time-points (p > 0.2). Infarct transmural extent did not correlate with strain (p > 0.05 at each time point). In multivariable logistic regression, MO and IMH were the only significant independent predictors of attenuated 90-day infarct zone strain (p = 0.004, p = 0.011, respectively).

**Conclusions:**

Strain improves within the infarct zone overall following reperfusion with or without MO or IMH. Mid-myocardial and endocardial infarct contractility is diminished in the presence of MO, and further in the presence of IMH. MO and IMH are greater independent predictors of infarct zone contractile recovery than infarct volume or transmural extent.

## Background

Following reperfused acute myocardial infarction (AMI), the left ventricle (LV) undergoes structural alterations both within and outside of the area of infarction, referred to as LV remodeling. The pathophysiology of this process is complex, with multiple ultrastructural, metabolic and neurally mediated processes occurring in infarcted and remote myocardium [1]. In up to 30% of patients, coronary reperfusion is associated with microvascular obstruction (MO) [2], seen angiographically as ‘no-reflow’ [3]. MO has been associated with adverse prognosis, adverse LV remodeling and diminished recovery of LV function, independently of infarct size [4,5]. Reperfusion may also lead to intramyocardial hemorrhage (IMH) in the infarct core [6] via extravasation of blood through damaged endothelium [7]. Like MO, IMH is associated with adverse LV remodeling and adverse prognosis, independently of infarct size [8]. The mechanisms by which MO and IMH affect LV remodeling are poorly understood. Specifically, it is not known how they affect local remodeling and recovery in the infarct zone compared with remote myocardium.

Cardiovascular magnetic resonance (CMR) can non-invasively evaluate myocardial infarction, MO and IMH. Enhanced myocardium on late gadolinium enhancement (LGE) imaging correlates with infarction histologically [9], whilst a hypoenhanced infarct core on LGE corresponds to MO [10]. IMH has been assessed by T2-weighted (T2w) and T2* CMR [8,11-13]. In chronic infarction, increasing transmural extent of infarction with LGE imaging correlates with impaired recovery of contractile function after revascularization [9]. However, in AMI, LGE can overestimate infarct size [14]. Within enhanced myocardium on LGE, contractile activity has been demonstrated both by measures of strain at rest [15], and as contractile reserve with dobutamine [16]. Contractile function can also be measured by CMR, using myocardial tissue tagging, allowing a direct comparison of contractility and infract characteristics from CMR data. We sought to investigate how MO and IMH affect contractile function as measured by tissue tagging CMR in infarcted and remote myocardium acutely and late following AMI.

## Methods

This prospective study was undertaken in a single tertiary center. Other analyses from this study have been reported previously [17]. The study protocol was approved by the institutional research ethics committee and complied with the Declaration of Helsinki; all patients gave written informed consent. Patients with first AMI, revascularized by primary percutaneous coronary intervention (PCI) within 12 hours of onset of pain were included [8]. Myocardial infarction was defined by symptoms consistent with acute myocardial ischemia, with electrocardiographic ST-segment elevation or new onset left bundle branch block associated with a rise and/or fall in cardiac enzyme concentration. Exclusion criteria were previous MI or coronary revascularization, estimated glomerular filtration rate <30 ml/min/1.73 m^2^, cardiomyopathy, or contraindications to CMR. Patients with maximal circumferential extent of myocardial scar <4 mm, without scar on adjacent slices were deemed too small for accurate tagging analysis and excluded from the analysis. Clinical management (including use of aspiration catheters and glycoprotein IIb/IIIa receptor inhibitors) was at the discretion of the responsible physician, with the intention to reflect contemporary practice and guidelines. CMR results were not revealed to the clinical team. All patients were considered for beta-blockade, angiotensin converting enzyme inhibitors, statins, dual antiplatelet therapy, and cardiac rehabilitation.

### Image acquisition

All patients had CMR on a 1.5 T system (Intera CV, Philips Healthcare, Best, The Netherlands) within 3 days of their index presentation and at 7–10, 30 and 90 days post-AMI. The same CMR protocol was used for each of the visits. A stack of images covering the whole LV, with the same slice geometry, position and slice thickness were used for all sequences. Cine imaging used a steady state free precession (SSFP) pulse sequence (echo time (TE) 1.4 ms; repetition time (TR) 2.8 ms; flip angle 55°, spatial resolution 2 × 2 × 10 mm, ≥18 phases per cardiac cycle), covering the whole heart in parallel short axis slices. To minimize differing volume effects between image types, 10 mm slice thickness was used for all sequences. Tagged CMR used a complementary spatial modulation of magnetization (CSPAMM) pulse sequence (spatial resolution 1.67 × 1.67 × 10 mm, no gap, tag separation 8 mm, ≥18 phases, typical TR/TE 30/6 ms, flip angle 20°). T2w CMR used a dark-blood T2w short tau inversion-recovery fast spin echo sequence (TE 100 ms, TR 2x R-R interval, flip angle 90°, spatial resolution 1.43 × 1.43 × 10 mm). T2* images were obtained with a dual echo T2* gradient echo sequence (TE 4.6/9.2 ms, TR 12.3 ms, flip angle 30°, spatial resolution 1.43 × 1.43 × 10 mm). A dose of 0.2 mmol/kg of gadolinium-DTPA (dimegluminegadopentetate; Magnevist, Bayer, Berlin, Germany) was then administered using a power injector (Spectris, Solaris, PA). A short-axis LGE stack was acquired after 10 minutes (inversion recovery-prepared T1 weighted gradient echo, inversion time according to a Look-Locker scout, TR/TE 4.9/1.9 ms, flip angle 15°, spatial resolution 1.35 × 1.35 × 10 mm). For follow-up, care was taken to ensure similar slice positioning, by aligning the proximal border of the most basal slice of the short axis stack to the mitral valve annulus in end-diastole and comparing slice position to the index scan.

### Image analysis

Images were analyzed offline using commercial software (MASS 7.2; Medis, Leiden, The Netherlands and Tagtrack 1.8; Biomedical engineering, ETH Zurich, Switzerland). Infarct location was determined by CMR, according to standard guidelines [18]. In addition to the alignment of slices during image acquisition, we verified accurate alignment of serial scans by comparing features such as the presence and shape of papillary muscles. Left ventricular volumes and wall thicknesses were analyzed from SSFP cine-imaging. Infarcts and MO were measured from LGE images. Infarct was defined as an area of LGE ≥2 standard deviations (SD) above remote myocardium, and infarct volume estimation included any hypointense core. This cut-off was chosen for consistency with analysis of T2w images. MO was defined visually as the hypointense core within the infarcted zone and planimetered manually. Volumes of infarct and MO were calculated from planimetered areas across the whole LV stack by the modified Simpson’s method. The presence and extent of myocardial hemorrhage was assessed by combined analysis of T2w and T2* sequences [8]. On T2w images, areas with mean signal intensity more than 2 SD below the periphery of the area at risk (AAR) were considered to be hemorrhage [11,13]. On the T2* images, the presence of a dark core within the infarcted area by visual inspection of the images was used as confirmation of myocardial hemorrhage. Only when T2w and T2* images showed concordant findings was an area considered to represent hemorrhage.

**Figure 1:**
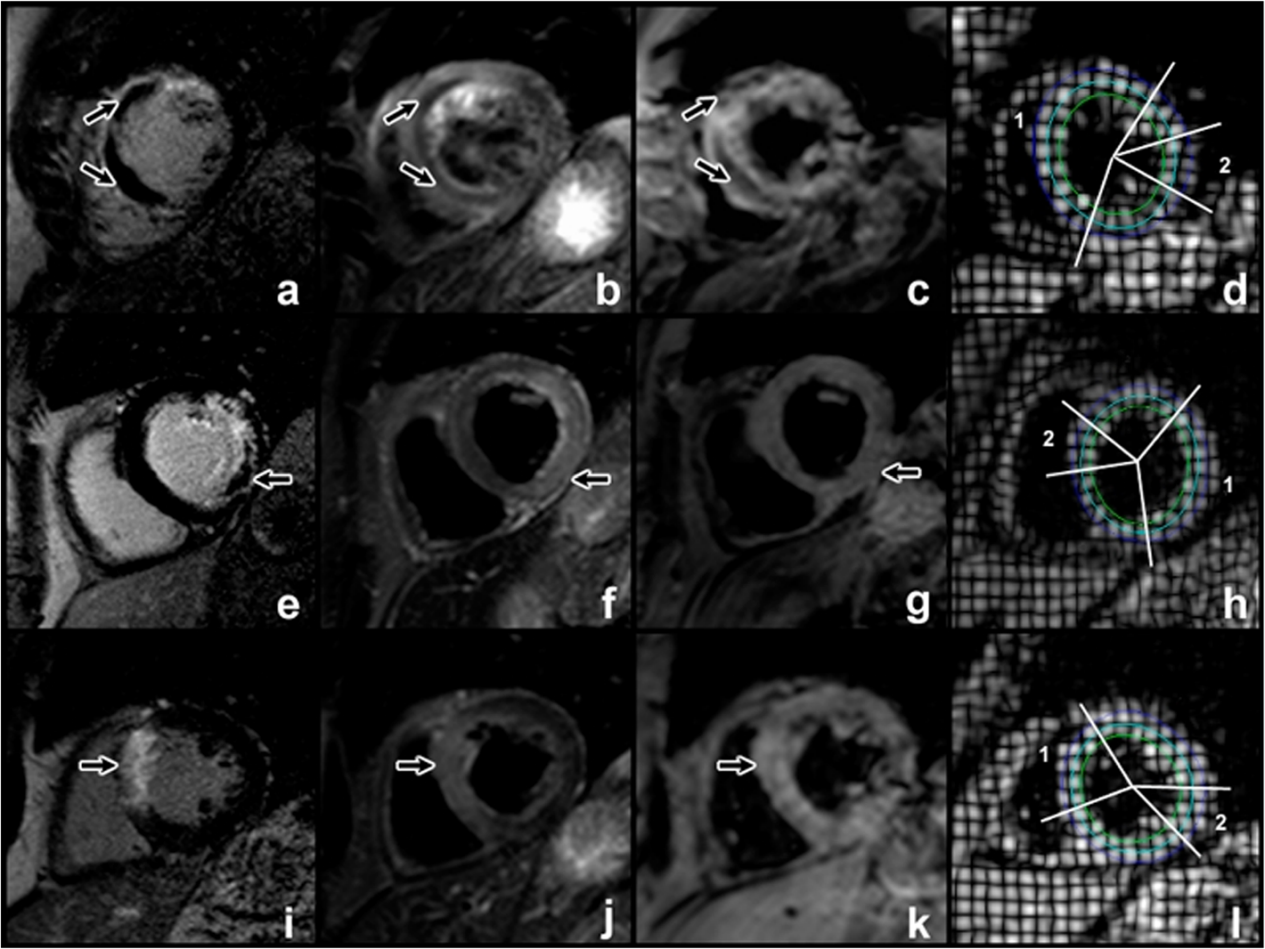
**Patient with MO and IMH (a-d).** LGE image demonstrates anterior and septal enhancement, corresponding to scar. The central hypoenhanced core corresponds to MO (**a**, arrowed). T2w (**b**) and T2* (**c**) imaging show central hypoenhancement (arrowed), indicative of IMH. Strain measurements in epicardial, mid-myocardial and endocardial tracks are measured (**d**) for infarcted (1) and remote (2) zones. A similar arrangement of images for infarction with MO but no IMH is shown (**e**-**h**). Note absence of central hypoenhancement in T2w and T2* sequences (arrowed). A patient without MO is shown (**i**-**l**).

For CSPAMM analysis, endocardial and epicardial borders were drawn by a semi-automated process, and a midline calculated automatically. To minimize partial volume effects, strain was measured in the single short axis slice which demonstrated maximal infarction on LGE imaging. One short axis slice per patient at each time point was analyzed for strain. In all cases, this slice was in the same position along the long axis for each of the time points in a given patient, and every slice had LGE on the adjacent slices. Circumferential Lagrangian strain was measured at endocardial, mid-myocardial and epicardial layers through the infarct and remote zones (Figure 1). Remote strain measurement was taken in a 30 degree arc of myocardium diametrically opposite to the infarct zone. No patient had LGE in remote myocardium. To minimize the effects of passive post-systolic shortening [19], strain was measured at end systole, taken as the phase at the time of end-systole on the corresponding SSFP cine slice.

Transmural extent of infarction was graded into quartiles at each time point from anonymized LGE images at the same position as the selected CSPAMM slice by consensus of two observers (AK and SP), blinded to the results of other sequences.

### Statistical analysis

Statistical analysis was performed using IBM SPSS® Statistics 19.0. Continuous variables were expressed as means ± SD. Correlation between T2w and strain data were derived using Spearman’s rank test; differences in strain and size measurements over time were evaluated using repeated measures analysis of variance (ANOVA); post-hoc testing was performed with the Bonferroni correction. Differences in transmurality quartiles over time were evaluated with the Friedman test. Normality for strain data was established using the Kolmogorov-Smirnov test. Differences in infarct pathophysiology at a single time point were evaluated using one-way ANOVA; post-hoc testing was performed with Tukey’s test. Univariable analyses were performed to identify predictors of reduced strain at 90 days. Variables with a probability value <0.1 in the univariable analysis were included in a multivariable analysis, which was based on a logistic regression model with a repeated measures variable (to adjust for non-independence of strain data). All statistical tests were 2-tailed; P values <0.05 were considered significant. Error bars for mean values denote standard error.

## Results

### Demographics

50 patients met the inclusion criteria. Two patients were excluded due to claustrophobia, 2 refused follow-up and 1 died before completing follow-up. In 6 other patients the tagging software failed to accurately track the tagged images due to artifact. Therefore 39 patients completed baseline and follow up scans and were included in the statistical analysis. Of these, 10 patients had tagging and T2w imaging at either day 2 or day 7, with 29 having these images at both these time points. All 39 had imaging at day 30 and day 90.

### Infarct characteristics

Patient data were divided into three groups for analysis; patients without MO or IMH, patients with MO but no IMH, and patients with both MO and IMH. No patient had IMH without MO. Patient characteristics were similar between the three groups (Table 1). Infarct characteristics are shown in Table 2.

**Table 1:** Patient characteristics

	**No MO or IMH (n = 17)**	**MO only (n = 8)**	**MO and IMH (n = 14)**	**p value**
Age, years	58 ± 10	55 ± 9	59 ± 8	0.70
Male	16 (94%)	8 (100%)	10 (71%)	0.12
Current smoker	9 (53%)	4 (50%)	7 (50%)	1.00
Hypertension	7 (41%)	3 (38%)	1 (7%)	0.08
Hypercholesterolemia	11 (65%)	3 (38%)	8 (57%)	0.50
Family history of premature heart disease	6 (35%)	3 (38%)	6 (43%)	0.91
Diabetes mellitus	0 (0%)	1 (13%)	1 (7%)	0.31
Pain to balloon time, min (median (IQR^*^))	205 (126)	194 (77)	210 (200)	0.93
Infarct territory				
Anterior	5	4	6	0.69
Inferior	11	3	6	
Lateral	1	1	1	
GP^†^IIb/ÍIIa inhibitor used	3 (18%)	3 (38%)	4 (29%)	0.64
TIMI flow pre-PCI ≥ 2	4 (24%)	0 (0%)	1 (7%)	0.25
TIMI flow post PCI				
Grade 2	1 (6%)	1 (11%)	0 (0%)	0.69
Grade 3	16 (94%)	8 (89%)	14 (100%)	
Peak CK^‡^ (U/l)	1234 ± 976	1711 ± 1442	2640 ± 1997	0.051

Data as mean ± SD or n (%). ^*^IQR interquartile range, ^†^*GP* glycoprotein, ^‡^*CK* creatine kinase.

**Table 2:** Infarct characteristics

**Days post AMI**		**No MO or IMH**	**MO only**	**MO and IMH**	**p value between groups**
2	Ejection fraction,%	45 ± 10^*^	42 ± 5^*^	38 ± 9^*^	0.10
	LV EDVi^†^, ml/m^2^	85 ± 14	96 ± 15^*^	93 ± 22	0.27
	LV ESVi^‡^, ml/m^2^	47 ± 14^*^	56 ± 14^*^	58 ± 20	0.16
	LV end-diastolic wall thickness, infarct zone, mm	8 ± 2^*^	9 ± 2	8 ± 2	0.72
	LV end-diastolic wall thickness, remote zone, mm	7 ± 1	8 ± 1	7 ± 1	0.78
	LV mass, g/m^2^	58 ± 15^*^	59 ± 11^*^	57 ± 19^*^	0.92
	LGE infarct volume, ml	21 ± 16^*^	32 ± 17	41 ± 20^*^	0.02
	Median LGE infarct transmural extent	75-100%	75-100%	75-100%^*^	0.10
	LGE MO volume, ml	-	3 ± 3^*^	4 ± 4^*^	0.41
90	Ejection fraction,%	51 ± 9^*^	50 ± 5^*^	42 ± 8^*^	<0.01
	LV EDVi, ml/m^2^	85 ± 19	90 ± 14^*^	100 ± 27	0.17
	LV ESVi, ml/m^2^	42 ± 17^*^	45 ± 11^*^	59 ± 23	0.04
	LV end-diastolic wall thickness, infarct zone, mm	7 ± 1^*^	7 ± 1	6 ± 1	0.13
	LV end-diastolic wall thickness, remote zone, mm	7 ± 2	7 ±1	7 ± 1	0.72
	LV mass, g/m^2^	55 ± 15^*^	56 ± 11^*^	52 ± 18^*^	0.82
	LGE infarct volume, ml	14 ± 10^*^	16 ± 7	29 ± 16^*^	<0.01
	Median LGE infarct transmural extent	75-100%	75-100%	50-75%^*^	0.73

Data as mean ± SD or n (%) unless indicated. ^*^Characteristics that are significantly different between day 2 and day 90 are indicated. ^†^*LV EDVi* Left ventricular end diastolic volume, indexed to body surface area, ^‡^*LVESVi* Left ventricular end systolic volume, indexed to body surface area.

**Figure 2:**
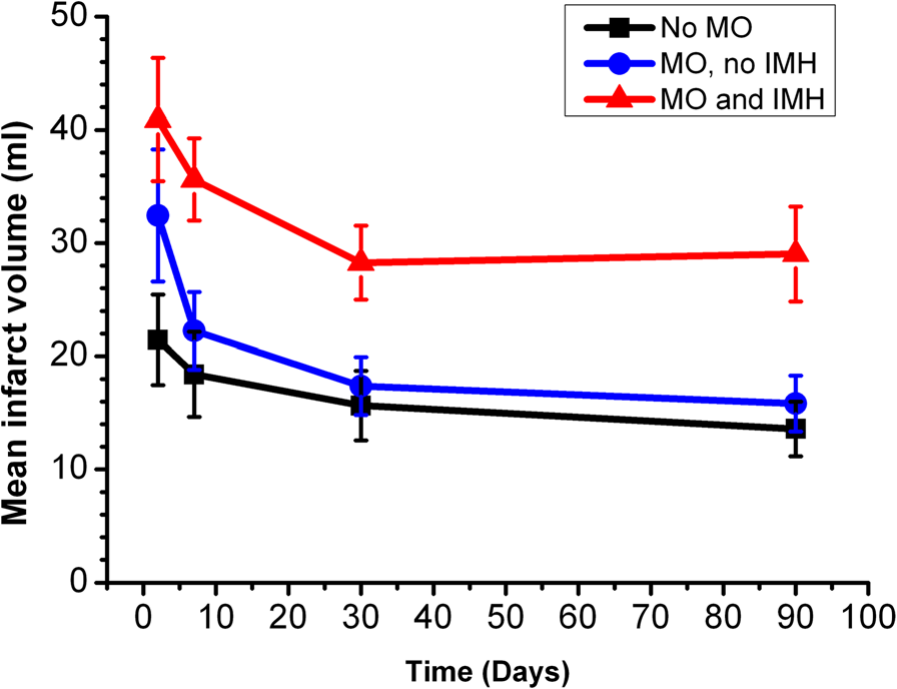
Mean infarct zone size at 4 time points post AMI, stratified by presence of MO and IMH.

Patients without MO or IMH had similar infarct size to patients with MO, but those with IMH had significantly larger infarcts than patients without IMH both at baseline (mean infarct volume 41 ± 20 ml vs. 21 ± 16 ml, p = 0.02) and 90 days (mean infarct volume 21 ± 16 ml vs. 14 ± 10 ml, p < 0.01). Infarct size decreased significantly over time in all three groups (no MO group, F = 7.5, p < 0.01; MO with no IMH group, F = 9.8, p < 0.01; MO and IMH group, F = 7.2, p = 0.01) (Table 2 and Figure 2). There was also a significant decrease in infarct transmural extent over time in the patients with MO and IMH (p < 0.01), but not in patients with MO and no IMH (p = 0.1) or without MO or IMH (p = 0.6) (Table 3). There was no significant difference in infarct transmural extent between the groups at any time point (p > 0.05 at each time point).

**Table 3:** Summary of changes in infarct characteristics between 2 and 90 days

**Variable**	**No MO or IMH**	**MO only**	**MO and IMH**
Infarct volume			
Ejection fraction			
Transmural extent of infarction			
Magnitude of infarct endocardial strain			
Magnitude of infarct mid myocardial strain			
Magnitude of infarct epicardial strain			
Magnitude of remote endocardial strain			
Magnitude of remote mid myocardial strain			
Magnitude of remote epicardial strain			

Arrows indicate direction of change. Horizontal arrow denotes no significant change.

### Effects of MO, IMH, infarct size and transmural extent on myocardial strain

**Figure 3:**
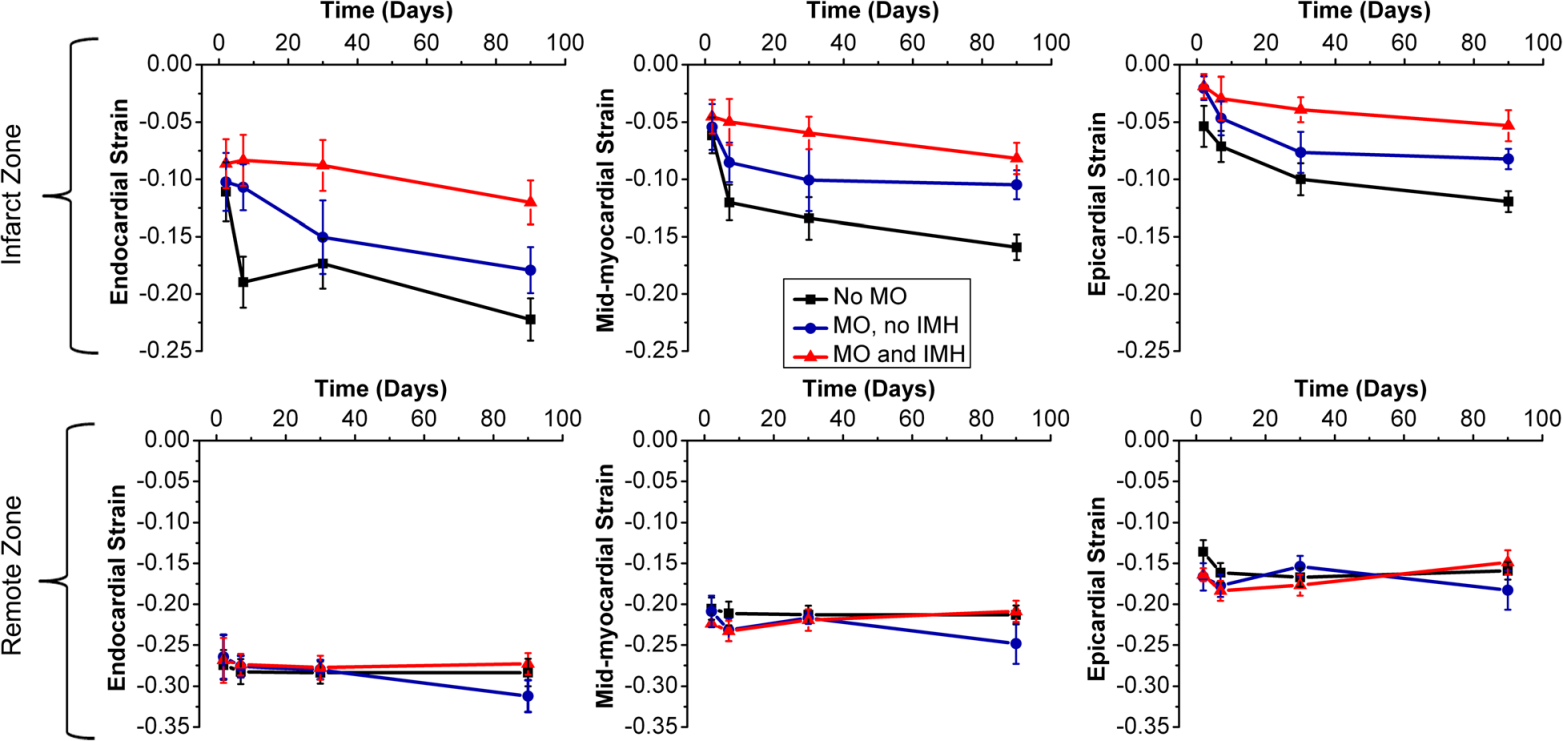
Endocardial, mid-myocardial and epicardial circumferential strain at 4 time points post AMI. Infarct zone (top row) and remote zone (bottom row) are shown.

Within the infarct zone, examining strain across all layers showed overall recovery with time (F = 44, P < 0.001). For individual layers, endocardial, mid-myocardial and epicardial strain recovered over the 4 time points (p ≤ 0.01 for each) for patients without MO or IMH. For patients with MO, regardless of the presence of IMH, there was no significant recovery of endocardial (F = 3.1, p = 0.05; F = 1.4, p = 0.3 in the absence and presence of IMH, respectively) or mid-myocardial strain (F = 2.1, p = 0.1; F = 2.9, p = 0.05 respectively), but epicardial strain recovered significantly (F = 7.7, p < 0.01; F = 3.3, p = 0.03 respectively; Figure 3).

Analysis of individual time points showed differences between infarcts with MO and IMH evolving over time. At day 2, there was no significant difference in infarct strain in endocardial, mid-myocardium or epicardial zones according to the presence of MO or IMH (F = 0.27, p = 0.8; F = 0.27, p = 0.8; F = 1.9, p = 0.2 for endocardial, mid-myocardial and epicardial strain, respectively). By day 7, there was significant difference in endocardial and mid-myocardial strain between the groups, but not epicardial strain (F = 6.9, p = 0.03; F = 4.3, p = 0.02; F = 1.9, p = 0.2 respectively). At day 30 and day 90, there were significant differences in endocardial, mid-myocardial and epicardial strain according to the presence of MO or IMH (day 30: F = 3.7, p = 0.03; F = 4.5, p = 0.02; F = 5.5, p < 0.01; day 90: F = 7.9, p < 0.01; F = 11, p < 0.01. F = 10, p < 0.01 respectively).

Remote myocardial strain was similar over time, and similar at each time point (p ≥ 0.2 for all) regardless of infarct characteristics (Figure 3).

At each time point, infarct zone endocardial strain was not associated with infarct transmural extent (F = 1.1, p = 0.3; F = 0.3, p = 0.8, F = 1.1, p = 0.3; F = 2.3, p = 0.1 for days 2, 7, 30 and 90 respectively). Endocardial strain was chosen as it was consistently within the infarct zone.

Univariable linear regression analysis (examining the variables in Table 4) showed that presence of MO, presence of hemorrhage and total infarct volume, but not infarct transmurality were significantly associated with decreased strain in the infarct zone at 90 days. Of these, the presence of MO and/or IMH, but not infarct volume, were significantly associated with strain on multivariable logistic regression analysis (Table 4).

**Table 4:** Predictors of decreased infarct zone strain in univariable and standard multivariable regression analysis

	**Univariable**	**Multivariable**
Variable	p value	p value
Age	0.40	…
Sex	0.84	…
Current smoker	0.04	…
Hypertension	0.13	…
Hypercholesterolemia	0.75	…
Family history	0.17	…
Diabetes	0.79	…
Onset to balloon time	0.18	…
Anterior AMI	0.34	…
TIMI flow grade before PCI > 0	0.69	…
TIMI flow grade after PCI < 3	0.46	…
Transmural extent of infarction >50%	0.12	…
Transmural extent of infarction 100%	0.58	…
Infarct volume (at 90 days)	0.001	0.07
Presence of MO only	<0.0001	0.03
Presence of MO + IMH	<0.0001	0.005

## Discussion

This study has found that contractile function as measured with CMR tissue tagging improves within the overall infarct territory following reperfused AMI with or without the presence of MO or IMH. However, this recovery was diminished in the presence of MO and further in the presence of IMH. Remote myocardial contractility did not change over time or with MO or IMH. The presence of MO and/or IMH was a stronger independent predictor of infarct zone contractile recovery than transmural extent of infarction or overall infarct volume.

There are limited existing data on how MO or IMH affect infarct zone contractile recovery. In canines, the extent of MO early post MI relates to reduced deformation and dysfunction of non-infarcted adjacent myocardium [20]. In humans, the relationship between infarction, MO and regional function has conflicting evidence. Rogers *et al.*[15], showed that enhanced myocardium on LGE recovered function with time, but hypoenhanced myocardium did not. Gerber *et al.*[21], found no significant difference in strain between hypoenhanced and enhanced segments 7 months following AMI. We have shown previously that IMH on T2 and T2* imaging post-AMI was the strongest predictor of adverse LV remodeling globally [8], and that strain recovers significantly within enhanced myocardium, even when accounting for scar zone remodeling [17].

In this study, patients with IMH had significantly larger infarcts than those without, as previously observed [8,22]. However, even when accounting for infarct size, patients with IMH had poorer infarct zone contractile function from day 7 onwards. The mechanisms by which MO and IMH confer reduced LV contractile function and adverse remodeling remain unclear [5,23], and are not simply explained by infarct expansion. Notably, we found no significant changes in remote strain, suggesting that MO and IMH affect contractility by processes in or around the infarct. Our findings of MO and IMH as the strongest independent predictors of attenuated infarct zone strain support this notion.

One possible interpretation of these findings is that circumferential motion is transmitted from a viable epicardial rim to the endocardium and that myocardial tagging by CMR detects this passive motion rather than active contraction. Epicardial strain recovery was consistently observed in all three groups and would support this interpretation. However, three points do not readily concord with this interpretation: 1) the transmural extent of infarction was similar across the three groups, and so one would expect that the magnitude of circumferential compression should be similar, 2) infarct endocardial strain did not show an association with infarct transmural extent and 3) patients with fully transmural infarction demonstrate strain recovery over time in the core of the infarct zone [17].

An alternative interpretation of our findings is that residual viable myocardium remains within the reperfused infarct zone and that the surviving myocytes are responsible for the evolving contractility over time in the infarct zone. The current literature on this issue is inconsistent. Most of the evidence suggests that enhanced zones on LGE imaging are entirely non-viable (reviewed in [24]). However, it is also known that enhancement on LGE CMR can overestimate infarct size acutely, and that the viable, edematous borderzone can show enhancement [25]. Histologically, preserved islands of viable myocytes have been shown to exist within the infarct zone [26]. In one study the mean myocyte fraction from sections of scar tissue defined by LGE imaging was as high as 62% [27].

What is less controversial is that the extent of damage within the infarct zone varies. Ultrastructural damage is more pronounced in areas of no-reflow [3,28] and IMH is associated with diminished healing within the infarct core, and altered inflammatory response [6]. It is possible therefore that any differences in contractile function and functional recovery between these pathologies reflect the variation in the proportion of residual viable myocytes following reperfusion that may not be apparent on qualitative LGE imaging. Alternatively, these structural differences may lead to differential transmission of epicardial contraction into the infarct zone depending on the presence of MO or IMH.

Patients without MO or IMH showed recovery of strain in the endocardial, mid-myocardial and epicardial infarct borders, but in patients with MO (with or without IMH), there was no significant recovery in endocardial and mid-myocardial areas. Furthermore, when examining the differences between the groups over time, endocardial and mid-myocardial contractile function was significantly different at day 7 whilst this was not apparent in the epicardial border until day 30. This accords with the wavefront theory of infarction, and that MO and IMH principally develop in the endocardium and mid-myocardium, with relative sparing of epicardial ischemia and infarction prior to reperfusion.

Hypoenhancement on LGE produces a conservative estimate of the extent of MO [29] compared with early gadolinium enhancement (at 2 min following contrast injection). We chose to define MO by LGE as it is considered to be of higher prognostic value [30]. In our study, findings on early and late gadolinium enhancement were similar and only one of the 16 patients with no MO on LGE showed MO on early enhancement imaging. The results and significant findings of the study were not changed by analyzing this patient in the MO group.

### Limitations

This study has limitations. The number of patients is relatively small, though sufficient to generate significant results and in keeping with other CMR studies in this demographic, where serial imaging including early post AMI is challenging. Five patients did not have strain and T2w imaging at baseline. It is possible that any MO/IMH may have resolved by day 7, although of 22 patients with MO at day 2, all but one had MO at day 7. We used a dual-echo T2* method in this study. Multi-echo techniques permit more reliable quantitative estimates of T2* but were not available to us at the time of this study. For this reason, we only performed a qualitative assessment of IMH size from T2* images. A variety of cut-offs to define LGE enhanced myocardium have been proposed in the literature; we chose 2SD to ensure consistency with T2 image analysis. Using concurrent LGE images for tagging at each time point may be affected by geometric changes in the LV or infarct zone for follow up scans, and a potential change in the position of myocardium that is sampled. Resolving three circumferential layers in remodeled infarcted myocardium may have been limited by the resolution of CMR, though the harmonic phase analysis software is able to produce a displacement map between tagging intersections to <1 mm [31], and the infarct zone did not thin in the vast majority of patients with timely reperfusion. End systolic strain may not account for infarct zone dyssynchrony, though this was not observed visually in any patient.

## Conclusions

The infarct zone following reperfused AMI demonstrates contractile recovery over time as measured by tissue tagging CMR. The presence of hypoenhancement on LGE, suggestive of MO, is associated with reduced functional recovery over time, affecting in particular endocardial and mid-myocardial functional recovery. Hypoenhancement on T2w and T2* imaging, suggestive of IMH, is associated with further reduction in infarct zone functional recovery. Both MO and IMH are independent predictors of impaired contractile recovery in the infarct zone, but neither independently affects remote myocardial contractility.

## Abbreviations

AMI: Acute myocardial infarction; CABG: Coronary artery bypass grafting; CMR: Cardiovascular magnetic resonance; CSPAMM: Complementary spatial modulation of magnetization; IMH: Intramyocardial hemorrhage; IQR: Interquartile range; LGE: Late gadolinium enhancement; LV: Left ventricle; MO: Microvascular obstruction; NS: Non-significant; PCI: Percutaneous coronary intervention; SD: Standard deviation; SSFP: Steady state free precession; T2w: T2 weighted.

## Competing interests

The authors declare that they have no competing interests. SP is funded by a British Heart Foundation fellowship (FS/1062/28409). SP and JPG receive an educational research grant from Philips Healthcare.

## Authors’ contributions

AK: Conception and design, analysis and interpretation of data, drafting of the manuscript; ANM: Conception and design, critical and intellectual revision of manuscript; PS: Interpretation of data, critical and intellectual revision of manuscript; MM: Critical and intellectual revision of manuscript; AU: Critical and intellectual revision of manuscript; JPG: Interpretation of data, critical and intellectual revision of manuscript; SP: Conception and design, interpretation of data, drafting of manuscript. All authors have given approval of this manuscript for publication.
